# SCAR Marker for Identification and Discrimination of *Commiphora wightii* and* C. myrrha*

**DOI:** 10.1155/2016/1482796

**Published:** 2016-03-16

**Authors:** Pramod Kumar Sairkar, Anjana Sharma, N. P. Shukla

**Affiliations:** ^1^Centre of Excellence in Biotechnology, M. P. Council of Science & Technology, Bhopal, Madhya Pradesh, India; ^2^Bacteriology Laboratory, Department of Post Graduate Studies & Research in Biological Science, Rani Durgawati University, Jabalpur, Madhya Pradesh, India; ^3^Madhya Pradesh Pollution Control Board, Bhopal, Madhya Pradesh, India

## Abstract

Commercially important* Commiphora* species are drought-tolerant plants and they are leafless for most of the year. Therefore, it is necessary to develop some molecular marker for the identification. Intended for that, in the present study, species-specific, sequence-characterized amplified regions (SCAR) markers were developed for proficient and precise identification of closely related species* Commiphora wightii* and* C. myrrha*, which may ensure the quality, safety, and efficacy of medicines made from these plants through adulterous mixing of these plants. Two species-specific RAPD amplicons were selected, gel-purified, cloned, and sequenced after screening of 20 RAPD primers. The sequence of 979 and 590 nucleotides (Genebank accession numbers K90051 and K90052) was used for development of 4 SCAR markers, namely, Sc1P, Sc1Pm, Sc2P, and Sc2Pm. Out of them, the Sc1Pm was specific for* C. wightii*, while Sc2P discriminated both the* Commiphora* species. These markers are first reported and will be useful for rapid identification of closely related* Commiphora wightii* and* C. myrrha* species.

## 1. Introduction

*Commiphora* spp. of the family* Burseraceae* is being used as a medicinal plant since ancient times and now rated as an endangered plant species [1]. They are found in the arid to semiarid regions of the world, including the deserts of India, Pakistan, Africa, and Saudi Arabia, while in India, it is found in Rajasthan, Madhya Pradesh, Gujarat, Tamilnadu, Orissa, and Karnataka. About 185 species of* Commiphora* were found worldwide, out of them* C. wightii* (synonym* C. mukul*),* C. agallocha*,* C. stocksiana*,* C. berryi*, and* C. myrrha* were found in India [2, 3]. In earlier studies about the flora of India, the “Guggul” plant was known as* Commiphora mukul* (Hook ex Stocks) Engl. or* Balsamodendron mukul* (Hook ex Stocks). Finally, it was named as* C. wightii* (Arn.) by Bhandari in 1964.

*C. wightii* was well-documented medicinal plant since 3000 years ago [4], having exciting biological activities like being anti-inflammatory, antimicrobial, hepatoprotective, muscle relaxing, antiarthritic, hypolipidemic, hypocholesterolemic, antiobesity, antioxidant, antimalarial, antimycobacterial, antischistosomal, larvicidal, and mollucidal [2, 3, 5–19].

*C. wightii* contains a bitter gum known as Guggul (Myrrh) in stems and leaves. The yellowish gum oozes upon making an incision and solidifies in the hot environment to a hard brownish resin. Guggul is medicinally important and is used in the treatment of hypercholesterolemia and cardiovascular diseases [9, 20]; it is also shown to have anticancerous activity [21]. The extract of gum Guggul, as gugulipid, guggulipid, or guglipid, is reported as a folk remedy in the Unani and Ayurvedic system of medicine. Two trans-isomers of Guggulsterone, namely, Guggulsterones E and Z, were reported in gum Guggul as important active steroid which are used as cholesterol-lowering agents. The pharmacological properties associated with gum Guggul include anti-inflammatory, antibacterial, anticoagulant, antirheumatic, COX inhibitory, and hypolipidemic activities that are mostly due to the presence of these steroids [22, 23]. In 1986, Guggul lipids were granted approval in India for marketing as a lipid-lowering drug [24]. Several products of standardized formulations of* C. wightii* were already in human use as cholesterol-lowering agents [22, 25].

*Commiphora* species have been called “taxonomically difficult,” because of being drought-tolerant plants and they are leafless for most of the year [26]. There is resemblance of gum Guggul with gum resin of other species within and outside of the genus, which make high risk of adulteration in commercial samples either deliberately to get more profit or accidentally. Therefore, it is important to validate the* C. wightii* plants and their gum Guggul in commercial samples due to its various pharmacological significances [27]. Many types of markers, namely, morphological, biochemical, and DNA based molecular markers, are commonly used in the identification of species [28]. Molecular markers were used in the identification of species and individual, their origin, and difference at the molecular level in between them [29]. During the last few decades, the use of molecular markers, revealing polymorphism at the DNA level, has been playing an increasing part in plant biotechnology and their genetic studies. These DNA based markers are differentiated into two types: first is non-PCR based RFLP and second is PCR based markers (RAPD, AFLP, SSR, SNP, etc.) [30]. RAPD is a PCR-based technology, based on enzymatic amplification of target or random DNA segments with arbitrary primers. The main advantage of RAPDs is that they are quick and easy to assay, had no sequence data required for primer construction, randomly distributed throughout the genome, and had a dominant nature [31]. However, RAPD marker is not suitable for the species identification, because of their low reproducibility and dominant nature [32]. A RAPD marker can be converted into a codominant and reproducible marker, that is, Sequence-Characterized Amplified Region (SCAR), which may be applicable for authentication of species.

Looking upon these problems, it is necessary to develop some molecular marker for the identification of* C. wightii*. In the present study, an attempt has been made for the development of SCAR markers for* C. wightii*.

## 2. Materials and Methods

Total 28 accessions of two different species of* Commiphora*, that is,* C. wightii* (17) and* C. myrrha* (11), were collected from Bhopal, Obaidullaganj (Madhya Pradesh), Akola (Maharashtra), Anand (Gujarat), and Jaipur (Rajasthan), and conserved at MPCST Human Herbal Health Care Garden, Bhopal.

### 2.1. Selection of RAPD Primers and Amplicons

Genomic DNA was isolated from fresh young stem* C. wightii* and* C. myrrha* using the method of Sairkar et al. (unpublished). The yield of DNA was measured using a NanoDrop UV-Spectrophotometer (ND-1000). Genomic DNA was amplified by the 20 primers (Table 1). A cocktail of 40 *μ*L reaction volumes was made with 20 *μ*L, 2x red dye PCR mix (Merck), 1 *μ*L primer (10 pM), and 1 *μ*L template DNA (25 ng/*μ*L) and amplification was performed on the gradient automatic thermal cycler (Eppendorf) following Sairkar et al. [33]. The PCR products were separated electrophoretically on 1.5% agarose gel at 5–10 volts/cm of the gel and visualized by ethidium bromide. The specific amplicon, which discriminates between* C. wightii* and* C. myrrha, was* selected and processed for the development of SCAR marker.

### 2.2. Cloning of Selected RAPD Amplicon

The selected amplicons were eluted using Medox-Easy Spin Column Cleanup Minipreps kit and ligated with the TA cloning vector (pGEM5Z, Promega). The ligated TA vector was transformed into competent cells of* E. coli* (DH5*α*), which was prepared using single step ultracompetent cell preparation kit (Medox). The first selection of recombinant clones was based on developed blue and white colonies on LB (Luria Burtani) agar plates containing 0.5 mg/mL ampicillin, 24 *μ*g/mL IPTG, and 30 *μ*g/mL X-gal. The plasmid of white and blue colonies was isolated through Medox-Easy ultrapure spin column plasmid DNA minipreps kit. Three selection steps, that is, clone retardation, restriction digestion, and amplification of plasmid, were adopted to identify positive insert within the plasmid. In retardation step, plasmids were separated electrophoretically to observe the presence of insert within plasmid, while in restriction digestion, plasmids were digested with PvuII enzyme for insert release. In the final step, the plasmids were amplified through the PCR reaction using 50 *μ*L that consist of 25 *μ*L 2x red dye PCR mix (Merck), 1 *μ*L each of forward and reverse M-13 primers (10 pM each), 1 *μ*L of plasmid DNA (25 ng/*μ*L) with a PCR profile of 94°C for 12 minutes, 30 cycles of 30 seconds at 94°C, 30 seconds at 55°C and 45 seconds at 72°C, and final extension on 72°C at 10 minutes using the gradient automatic thermal cycler (Eppendorf).

### 2.3. Designing and Screening of SCAR Marker

Plasmid having desired amplicon was sequenced by Aristogene Pvt. Ltd., Bangalore, India, using M13 reverse and forward sequencing primers and consensus sequence of amplicons was developed. The homology search of consensus sequences was performed by the NCBI BLAST tool. The primer pairs were designed for these sequences by using PRIMER 3 software [34] and used as a candidate for SCAR primer. Four accessions of each species of* C. wightii* and* C. myrrha* were amplified through these primer pairs (synthesized by Aristogene) with a cocktail of 40 *μ*L containing 20 *μ*L of 2x red dye PCR mix (Merck), 1 *μ*L of each of the SCAR primer pair (10 pM each), and 1 *μ*L of template DNA (25 ng/*μ*L). Amplification was performed on the gradient automatic thermal cycler (Eppendorf) with PCR conditions: 94°C for 5 minutes, 30 cycles of 30 seconds at 94°C, 30 seconds at 58°C and 1 minute at 72°C, and final extension on 72°C at 10 minutes. Among the all designed primer pairs, suitable primer pair was selected which discriminate the both species of* Commiphora* and further screened in all the accessions for validation of SCAR marker.

## 3. Result

### 3.1. Identification of RAPD Primer and Amplicon

**Figure 1:**
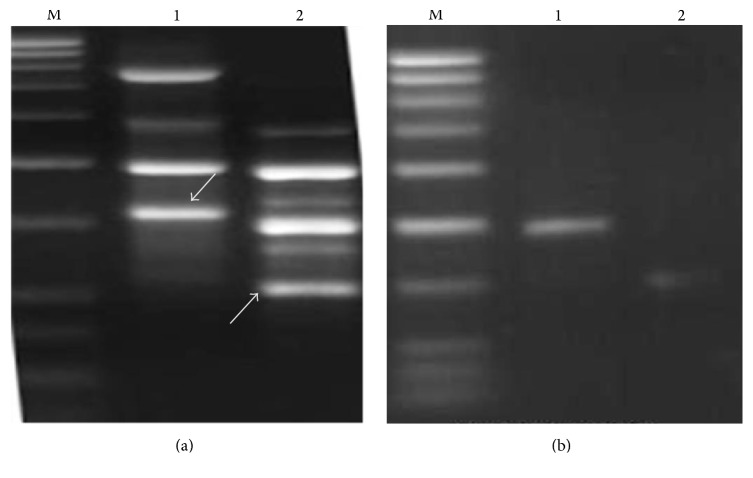
Selection and elution of desired amplicon. (a) PCR product of sample* Commiphora wightii* on low melting agarose gel. Lanes 1 and 2 amplified by primers OPD-02 and OPD-08. (b) Eluted desired amplicon run on agarose gel. Lanes 1 and 2, fragment sizes 1 kb and 0.6 kb, respectively.

Out of 20 RAPD primers, 1 kb amplicon of OPD-02 and 0.6 kb amplicon of OPD-08 discriminate both* Commiphora* species as it was present only in* C. wightii* accessions (Figure 1(a)). Due to specificity of these amplicons, they were cloned, sequenced, and used for SCAR marker development. These bands were elected from agarose gels and gel electrophoresis revealed that they were appropriate for cloning (Figure 1(b)).

### 3.2. Cloning and Selection of Positive Clone

**Figure 2:**
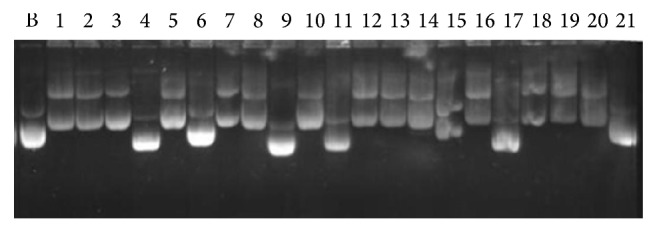
Retardation checking by plasmid run on agarose gel; Lanes 1 to 21: positive cloned plasmids 1 to 21, B: negative cloned plasmid isolated from blue colony.

**Figure 3:**
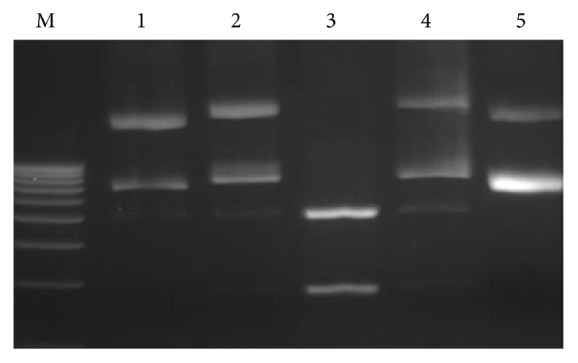
Insert release, digested plasmids by restriction endonuclease PvuII run on agarose gel, plasmid of Lanes 1, 2, 4, and 5 realised 1 kb fragment after digestion.

**Figure 4:**
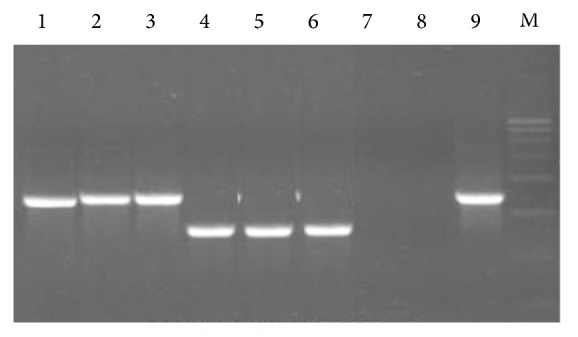
Amplification of plasmid by vector primers (M-13). Lane 1, 2, 3 and 9 produced 1 kb fragment while lanes 4, 5, and 6 produced 0.6 kb fragment.

White colony of competent cells (*E. coli*) having T vector with 1 kb and 0.6 kb insert was undertaken for plasmid isolation and three selection criteria were performed for the conformation of positive clone. The screening for retardation checking reveals that 17 positive plasmids for 1 kb insert and 5 positive plasmids for 0.6 kb may have proper insert (Figure 2). These positive plasmids were digested with the restriction endonuclease (PvuII) for insert release. A total of 6 positive plasmids of 1 kb insert and 4 positive plasmids of 0.6 kb insert release their respective insert fragment (Figure 3). In the third stage of selection, 4 positive plasmids for 1 kb insert 3 positive plasmids for 0.6 kb insert were finalized for sequencing after amplify with M-13 primer (Figure 4).

### 3.3. Sequencing and* In Silico* Application

**Figure 5:**
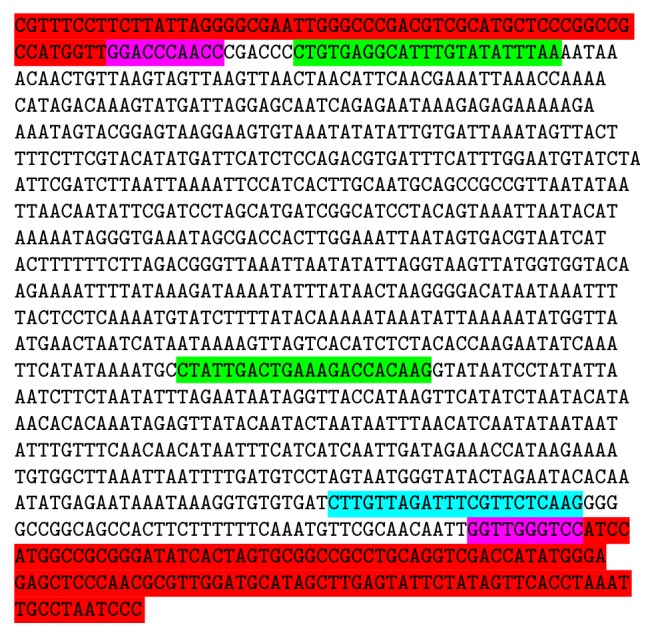
Consensus sequence (979 bp) of 1 kb fragment (Genebank ID K90051). Red highlights: vector sequence. Green highlight: SCAR primer region. Blue highlight: second reverse SCAR primer. Pink highlight: RAPD primer region.

**Figure 6:**
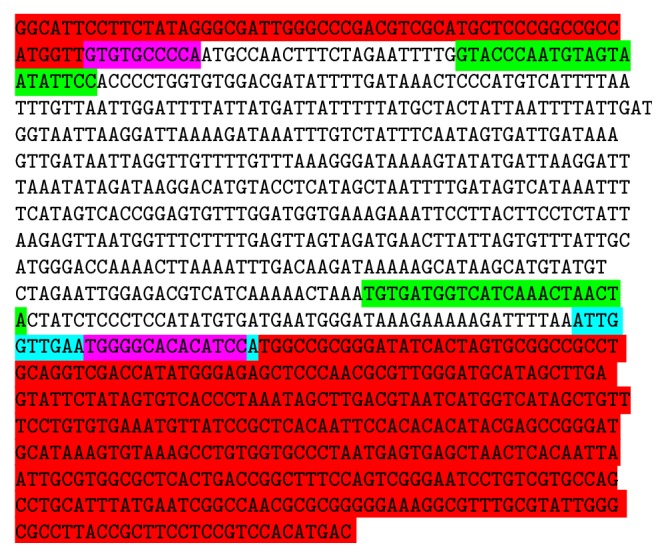
Consensus sequence (590 bp) of 0.6 kb fragment (Genebank ID K90052). Red highlights: vector sequence. Green highlight: SCAR primer region. Blue highlight: second reverse SCAR primer. Pink highlight: RAPD primer region.

The clones were sequenced and 979 bp and 590 bp consensus sequences were formed for 1 kb insert 0.6 kb insert, respectively (Figures 5 and 6). The BLAST search was performed for the obtained sequences and no significant homologous sequence was found in the NCBI database. This DNA sequences were deposited in the NCBI gene bank database with accession numbers K90051 and K90052. Two candidate SCAR primer pairs for each DNA sequences were designed, that is, primers Sc1P and Sc1Pm from 979 bp sequences and primer Sc2P and Sc1Pm from 590 bp sequences (Tables 1 and 2). These primers were deposited in the NCBI Prob database with accession number Pr031905450 to Pr031905453.

### 3.4. Development of SCAR Marker

**Figure 7:**
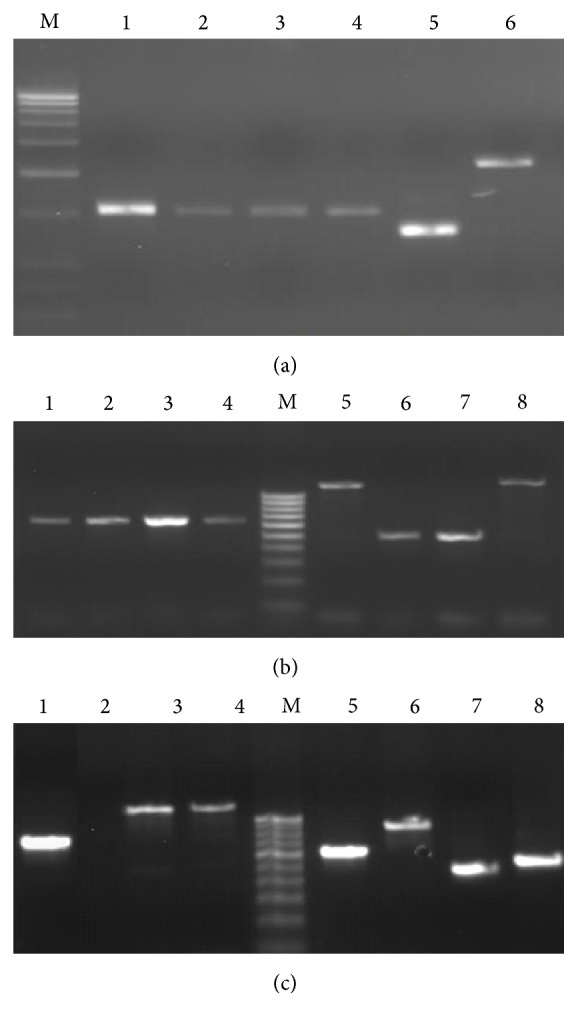
Screening of SCAR primers. (a) Lanes 1 to 2:* C. myrrha* and Lanes 3 to 4:* C. wightii* amplified by primers Sc1P; Lanes 5 to 6:* C. myrrha* and* C. wightii*, amplified by primers Sc2P. (b) Lanes 1 to 2,* C. myrrha* and Lanes 3 to 4,* C. wightii* amplified by primers Sc1P; Lanes 5 to 6,* C. myrrha* and Lanes 7 to 8,* C. wightii*, amplified by primers Sc2P. (c) Lanes 1 to 4,* C. myrrha* amplified by primers Sc1P, Sc1Pm, Sc2P, and Sc2Pm; Lanes 5 to 8,* C. wightii* amplified by primers Sc1P, Sc1Pm, Sc2P, and Sc2Pm.

The candidate SCAR primer pairs were screened with three accessions of each of* C. wightii* and* C. myrrha* which revealed that the primer Sc1Pm is highly specific for* C. wightii* and amplified 910 bp amplicon, while primer Sc1P had a similar banding pattern in all the samples. Primer Sc2P discriminated both the* Commiphora* species as it gave 491 bp amplicon for* C. wightii*, and 1200 bp for* C. myrrha*, while primer Sc2Pm gives 491 and 570 bp amplicon for* C. wightii* and* C. myrrha*, respectively (Figure 7).

**Figure 8:**
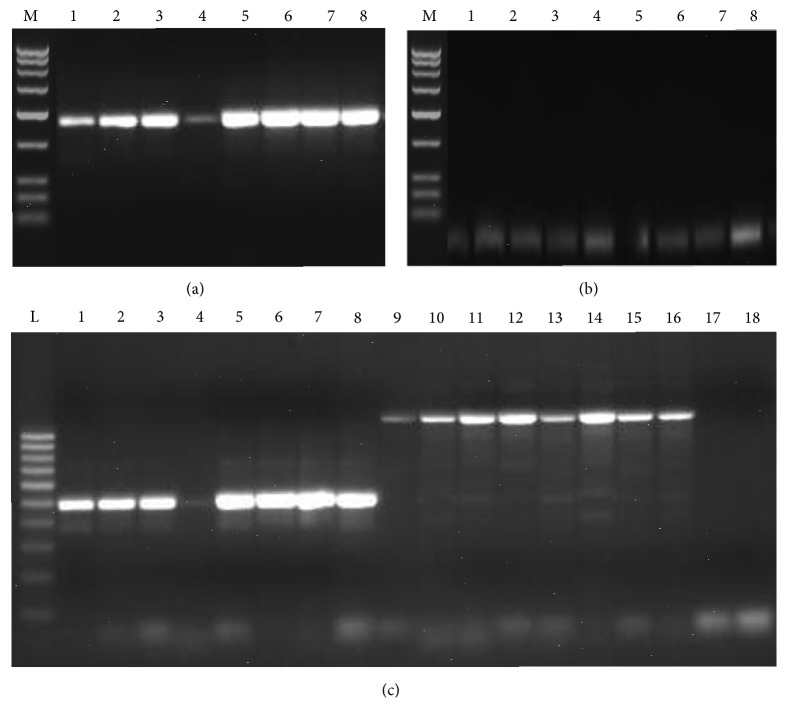
Screening of SCAR primers. (a) Lanes 1 to 8, accessions of* C. wightii* amplified by primers Sc1Pm. (b) Lanes 1 to 8, accessions of* C. myrrha* amplified by primers Sc1Pm. (c) Lanes 1 to 8, accessions of* C. wightii* and Lanes 9 to 16, accessions of* C. myrrha* amplified by primers Sc2P. M = low range ruler (3000, 2500, 2000, 1500, 1000, 600, 300, 200, and 100 bp). L = 100 bp ladder (1000, 900, 800, 700, 600, 500, 400, 300, 200, and 100 bp).

Based on the above results, primers Sc1Pm and Sc2P were authenticated through amplification of eight accession of each species, that is,* C. wightii* and* C. myrrha* (Figure 8). The similar results were observed during this screening as they were discriminated both the species of* Commiphora*.

## 4. Discussion

Identification of plants at the species level traditionally is a feverish job and needs special care during identification. This work usually needs to be a specially trained expert, after that many human errors were observed. To overcome this problem, in the early 1990s many specific molecular identification technologies were popular which are more reliable [35, 36]. Nowadays molecular taxonomists are engaged in preparation of the nucleotide sequence of a short DNA fragment for all living species on earth, which is called DNA barcodes [37–39].

Molecular markers allow the detection of specific DNA sequence differences between tests of individuals of an organism [40]. DNA markers are unlimited in number and are not affected by environmental factors and developmental stages of the plant [41]. The discovery of PCR technology changed the entire molecular biology and a single random oligonucleotide primer (10-bp long) was discovered in 1990 as a universal marker technology called RAPDs [42]. The main advantages of RAPD markers are the following: they are universal and cost-effective and for application of these markers they did not need any genetic information of the target organism and they can map almost completed genomic DNA of the target organism [43]. However, each method analyses different aspects of DNA sequence variation and different regions of the genome. RAPD and AFLP markers appear to frequently target repetitive regions of the genome.

The presence of polysaccharides, polyphenols, and other secondary metabolites in the leaves of* Commiphora* species creates complications in the DNA process. Haque et al. 2008 [44] and Samantaray et al. [45] used various methods and described the process for DNA isolation. Their isolated DNA showed good PCR amplification; therefore, it can further be used in molecular downstream applications. Molecular variations among accessions collected from different localities of Rajasthan and Gujarat were described by Suthar et al. [46]. Intraspecific variation in* Commiphora wightii* populations was described by Haque et al. [47] using Internal Transcribed Spacer (ITS1-5.8S-ITS2) Sequences while Harish et al. [48] studied genetic variations on accessions collected from Indian Thar Desert using RAPD and ISSR markers. Molecular variations among different biotypes of* Commiphora wightii* were done by Vyas and Joshi in 2015 [49] using RAPD markers. Genetic variability among the* C. wightii* germplasm collected from Rajasthan and Haryana was studied by Kulhari et al. [50]. Samantaray et al. [51] used sixty different random decamer primers and identified three primers which produced specific fragment in the female plant of* C. wightii* but failed to do so from the male plant DNAs. Their finding was helpful for the breeding practice of* C. wightii* and our SCAR markers may be useful for identification of* C. wightii* at species level.

The developed SCAR markers by us were used for identification of* C. wightii* and discrimination among* C. wightii* and* C. myrrha*. SCAR markers maybe are developed using sequence of RAPD fragments which are characterized by many advantages, including their specificity, low cost, ease, fast use, reproducibility, abundance, and being polymorphic in nature targeting specific regions of the genomes [52–54] employed with success in plant and animal species identification [30, 55–58].

In this study, RAPD amplicons were selected for cloning, sequencing, and final development of SCAR markers. Specific characters of RAPD markers entice researchers and usually SCAR markers have been developed from RAPD amplicons [58–61]. Amplicon of other fingerprinting methods like AFLP [62–64] and ISSR (Inter Simple Sequence Repeat) [52] was also used to develop SCAR markers.

Developed Sc1Pm marker in this study produced a 910 bp amplicon with* C. wightii*, while in other samples no amplification was observed. These results revealed that this SCAR marker might be used in identification and authentication of* C. wightii*. Many reports are available in which SCAR markers have been used for authentication of medicinal plant species like* Panax ginseng* [65], bent-grass [66], Bamboo [67],* Piper longum* [68],* Artemisia princeps* and* A. argyi* [69],* Phyllanthus emblica* [70], strawberry [71]* Jatropha curcas* [72, 73],* Ganoderma lucidum* [74],* Pueraria tuberosa* [75],* Dendrobium candidum* [76],* Sinapis arvensis* [77],* Cornus officinalis* [78], and* Scrophularia ningpoensis* [79]. Three SCAR markers of* Phyllanthus* species were developed from three specific RAPD sequences that can identify and differentiate the morphologically similar* Phyllanthus* species [80].

SCAR markers have been also developed for breeding programs of crops like Rice [81],* Citrus tristeza* [82],* Brassica napus* L. [83], Grapevine [84], Wheat [85], Buckwheat [86], Grape [87], Barley [88],* Atractylodes japonica* and* A. macrocephala* [89],* Diplocarpon rosae* [90],* Puccinia coronata* [91],* Puccinia striiformis* [92],* Thinopyrum elongatum* [93],* Liriope* and* Ophiopogon* [94],* Medicago sativa* [95],* Triticum turgidum* [96], and* Miscanthus sacchariflorus* [97].

Our marker Sc2P produced a prominent amplicon of 491 bp in the* C. wightii*, and 1.2 kb in the* C. myrrha* while other plant samples did not show amplification. The result revealed that this SCAR primer might be used for the discrimination among* C. wightii* and* C. myrrha*. Only few reports are present with a single primer discrimination among two closely related species.

## Figures and Tables

**Table 1:** Sequence of SCAR markers designed using 1 kb amplicon.

Name of SCAR	Name of fragments	Sequence (5′-3′)	Total length	Temp	Size (bp)
Sc1P	Sc1P (F)	CTGTGAGGCATTTGTATATTTAA	23 bases	60°C	631
	Sc1P (R)	CTTGTGGTCTTTCAGTCAATAG	22 bases	62°C	

Sc1Pm	Sc1P (F)	CTGTGAGGCATTTGTATATTTAA	23 bases	60°C	910
	Sc1Pm (R)	CTTGAGAACGAAATCTAACAAG	22 bases	60°C	

**Table 2:** Sequence of SCAR markers designed from 0.6 kb amplicon.

Name of SCAR	Name of fragments	Sequence (5′-3′)	Total length	Temp.	Size (bp)
Sc2P	Sc2P (F)	GTACCCAATGTAGTAATATTCC	22 bases	60°C	491
	Sc2P (R)	TAGTTAGTTTGATGACCATCACA	23 bases	62°C	

Sc2Pm	Sc2P (F)	GTACCCAATGTAGTAATATTCC	22 bases	60°C	570
	Sc2Pm (R)	GTGTGCCCCATTCAACCAAT	20 bases	60°C	
